# Health system resilience: Lebanon and the Syrian refugee crisis

**DOI:** 10.7189/jogh.06.020704

**Published:** 2016-12

**Authors:** Walid Ammar, Ola Kdouh, Rawan Hammoud, Randa Hamadeh, Hilda Harb, Zeina Ammar, Rifat Atun, David Christiani, Pierre A Zalloua

**Affiliations:** 1 Ministry of Public Health, Beirut, Lebanon; 2 Faculty of Health Sciences, American University of Beirut, Beirut, Lebanon; 3 Harvard T.H. Chan School of Public Health, Harvard University, Boston MA, USA; 4 School of Medicine, Lebanese American University, Beirut, Lebanon

## Abstract

**Background:**

Between 2011 and 2013, the Lebanese population increased by 30% due to the influx of Syrian refugees. While a sudden increase of such magnitude represents a shock to the health system, threatening the continuity of service delivery and destabilizing governance, it also offers a unique opportunity to study resilience of a health system amidst ongoing crisis.

**Methods:**

We conceptualized resilience as the capacity of a health system to absorb internal or external shocks (for example prevent or contain disease outbreaks and maintain functional health institutions) while sustaining achievements. We explored factors contributing to the resilience of the Lebanese health system, including networking with stakeholders, diversification of the health system, adequate infrastructure and health human resources, a comprehensive communicable disease response and the integration of the refugees within the health system.

**Results:**

In studying the case of Lebanon we used input–process–output–outcome approach to assess the resilience of the Lebanese health system. This approach provided us with a holistic view of the health system, as it captured not only the sustained and improved outcomes, but also the inputs and processes leading to them.

**Conclusion:**

Our study indicates that the Lebanese health system was resilient as its institutions sustained their performance during the crisis and even improved.

Lebanon is a country in the Middle East with a population of 4.2 million people [1]. Since the 1970s, Lebanon has endured repeated shocks to its health system, including wars, massive population displacement, economic downturns and political instability, which have produced a country that has been without a president for two years [2]. In addition to the persistent political instability, Lebanon has been housing half a million Palestinian refugees since 1948. The most immediate challenge facing Lebanon, however, is the Syrian refugee crisis.

Since 2011, following the escalation of the devastating war in Syria, there has been a large and ongoing influx of Syrian refugees to Lebanon. By the end of 2015 the numbers of refugees had reached 1.5 million, in addition to 53 000 Palestinians returning from Syria [3,4]. The new refugees represent a 30% increase in Lebanon’s pre–crisis population, resulting in the highest refugee density of any country worldwide since 1980 [5]. The refugees from Syria have not been placed in formal camps, but are dispersed across Lebanon in houses among the Lebanese population, while 17% is residing in informal tented settlements [6].

The unprecedented influx of refugees has placed a considerable burden on the Lebanese government, society and economy, which are facing many other challenges. For example, while the Gross Domestic Product (GDP) of Lebanon grew by 8% in 2010, it fell sharply to 1.5% in 2013 [7], constraining the government’s ability to continue financing the expanding population needs in the presence of stagnant economic growth.

A refugee crisis of such a large magnitude is a severe shock to the health system, and threatens continuity of service delivery, destabilizing governance and limiting access to care [8]. To date, however, the Lebanese health system (**Box 1**) has been able to accommodate and adjust to the refugee crisis [11].

Box 1 **Lebanese health system – a brief overview**

The health system in Lebanon is a public–private partnership with multiple sources of funding and channels of delivery. Almost one half of the population is financially covered by the National Social Security Fund (NSSF), an autonomous public establishment or by other governmental (civil servants cooperative and military schemes) or private insurance. All those schemes provide financial coverage with variable patient copays. The non–adherents are entitled to the coverage of the Ministry of Public Health (MoPH) for secondary and tertiary care at both public and private institutions. Palestinian refugees are covered through the United Nations Relief and Work Agency for Palestinian refugees (UNRWA) for their health care services [9].

Although the MOPH does not cover ambulatory care services, it provides in kind support to a national network of primary health care (PHC) centers all over Lebanon [2]. The centers provide consultations with medical specialists at reduced cost, as well as medicines for chronic illness and vaccines funded by the ministry of health [2].

Around 68% of the primary health care centers in the national network are owned by NGOs while 80% of hospitals belong to the private sector [2]. The strong presence of the private sector in service delivery has led to an oversupply of hospital beds and technology [2]. While there is an oversupply of physicians, there is a shortage of nurses [10].

Resilience is the ability of a health system to sustain or improve access to health care services while ensuring long–term sustainability [12,13]. A resilient system has high tolerance to uncertainty and relies on a variety of resources in its response to shock [14,15]. Despite calls for strengthening policy capacity in this important area [16,17] resilience of health systems to external and internal shocks remains understudied [18,19].

Lebanon is currently facing an acute crisis [20] due to an unprecedented influx of refugees from Syria with multiple health needs, which has placed a rapid and an unprecedented demand on the health system [21,22]. The extraordinary situation of the refugee crisis offers a unique setting to study resilience of Lebanon's health system to an external shock, combined with internal shocks due to economic and political instability.

## METHODS

The aim of this study is to assess the resilience of the Lebanese health system in the face of an acute and severe crisis and in the context of political instability.

While many conceptual frameworks for resilience exist [23] there is no unified definition of health system resilience, or an established method to measure it [24]. One framework offers 5 dimensions to assess the potential of health system resilience to an emerging crisis, but a standardized set of internationally accepted indicators for these dimensions have yet to be developed and tested empirically [25]. For the purpose of this study, we have used the following working definition of a resilient system: “a resilient system has the capacity to absorb change due to external or internal shocks, maintain original functions and ensure long–term sustainability” [26–29].

When studying the resilience of the Lebanese health system we drew on insights from studies of health systems that have faced refugee crises – studies which have considered the ability of a health system to maintain service delivery, prevent major outbreaks and sustain improvements in population level outcome indicators including utilization, service coverage, morbidity and mortality rates, as measures of success [30–33]. The indicators used in these earlier studies are in line with the definition of resilience we have used. This definition stresses the ability of a system to reorganize and adapt to change while maintaining original functions and ensuring long–term sustainability [26–28].

The study employs a case study approach and draws on data from multiple sources. We use an input–process–output/outcome model of a health system [30], where inputs, processes and outputs measure the capacity of the health system while outcomes measure its performance [30]. This allows for a comprehensive and holistic analysis of the Lebanese health system and offers enough flexibility to capture both the contextual characteristics of the system and factors in place during the acute crisis that have affected the health system response and resilience.

The study, which took place from January 2014 to July 2015, consisted of two main components: a literature review on resilience and how to measure it, and analysis of secondary data to document the impact of the refugee crisis and the health system response in Lebanon.

For the literature review, we undertook a search using the following databases: OVID Medline, the Cochrane Library, and Health Systems Evidence. In addition, we searched gray literature databases such as Reliefweb, MedNar, OAISTER, Open DOAR, PROSPERO and OpenGrey.

Local data were obtained from multiple sources, namely the Lebanese Ministry of Public Health database which included data on service utilization, human resources, immunization coverage, and epidemiological surveillance. We also used national health accounts data (that uses the System of Health Accounts 1.0) and maternal mortality observatory data. The MOPH information systems and the maternal mortality observatory data sets are designed to incorporate ongoing assessment and reporting related to displaced Syrians, including for immunization coverage, disease surveillance and utilization of health services in addition to maternal and child mortality. Other sources included statistics from the Lebanese Ministry of Finance, Bank of Lebanon and the Central Administration for Statistics (CAS), UN agency publications, World Bank assessments, and international and local NGOs publications.

## RESULTS

### Health system inputs and processes

**Human resources.** The fluctuating pattern in the number of physicians started before the Syrian Refugee crisis as a result of a mismatch in supply and demand, with persistent oversupply [34]. By contrast, the number of nurses working in Lebanese health system increased steadily and was not affected by the Syrian crisis [34]. The steady rate of increase in number of nurses occurred as a result of deliberate MOPH policies, such as the establishment of a career path for nurses, financing of training of more nurses by the Lebanese university, supporting the bridging between vocational and academic training, and increasing nursing wages in the public sector [2].

**Financing.** In 2013–14, there was no substantial change in patterns of public spending on health, the budget of the MOPH, and all public funds rose at the same rate of yearly increase as in the preceding years [1,35]. However, throughout the crisis, the levels of funding from international donors were erratic and far below the amounts required to meet the health needs of the refugees. For example, in 2013, less than 50% of funding requirement was met [36], declining in 2014 to 33% of the funding amount needed [4]**.** The funds from international donors are managed by United Nations (UN) agencies and are channeled through different international and local NGOs. The MOPH was not a recipient of these funds but worked with international entities to influence effective application of the funds to priority areas and populations.

Throughout the crisis, the Lebanese health system was able to sustain the level of financing of services at primary–, secondary– and tertiary–care levels. The MOPH contracts with primary health care centers were maintained. The MOPH was able to uphold and improve its contracting terms with private hospitals by including performance measures in the contracts to achieve required service volumes at specified quality levels. Additionally, all the public funds and private insurance companies continued to provide cover to their respective beneficiaries, notwithstanding delays in reimbursement.

Despite financial constraints, the MOPH managed to increase its expenditure on drugs, which helped to effectively meet the higher demand that arouse in recent years [1]. This expenditure of funds to increase expenditure on drugs was coupled with collaboration with different donors in order to direct external funds to priority areas.

For Syrian refugees, primary care has been partly subsidized by the United Nations High Commission for Refugees (UNHCR). However, for secondary care the financial assistance provided by UNHCR has been limited to vulnerable groups, and for life–threatening conditions with co–payments provided by refugees [36]. The limited financing of secondary care services has resulted in a major gap in service coverage, howeverleading to heavy financial burden on refugees seeking secondary and tertiary care services [36].

**Governance.** At the start of the crisis, there was no clear government policy regarding the displaced Syrian population. While the MOPH began to offer displaced Syrians the same immunization and schedule primary health care services offered to Lebanese citizens, UNHCR and other relief agencies sought to create their own delivery channels and their own mechanism financing coverage which operated in parallel to the existing health system. The parallel systems established by international agencies led to fragmentation and poor coordination of the health system response to the refugee crisis. In the absence of a clear government policy, the fragmentation of health system governance prompted the MOPH to call upon international agencies to consider a more integrated approach to planning, financing and service delivery by embedding refugee health care within the national health system. To develop an integrated approach, the MOPH established a steering committee that includes major international and local partners to guide the response. The steering committee, led by the MOPH, develops strategic plans and coordination mechanisms and monitors the response [4]. All partners in the refugee health response including MOPH, UN agencies, international and local non–governmental organisations (NGOs) held regular meetings and set up yearly response plans such as the “Lebanon crisis response plan”. These response plans detailed all funding sources, activities performed and coordination efforts. These plans were regularly updated and tracked, and the results were shared in dissemination workshops and on the websites of these partners. The inclusive model of governance, based on participation, transparency and accountability, was critical in mounting an effective emergency response and in creating health system resilience, and in establishing an effective surveillance system (**Box 2**).

Box 2 **Surveillance of refugees**

The registration of Displaced Syrians began in January 2012 by the UNHCR. By 6th May 2015, UNHCR had temporarily suspended new registrations as per Government of Lebanon's instructions. The highest influx occurred in 2013, with the number of Displaced Syrians reaching around 1 200 000 by end of 2014. The number of Displaced Syrians had declined by 31st October 2015 to 1 075 637.

In response to the rapid rise in demand for human health resources, the MOPH, in collaboration with UNHCR, WHO and UNICEF, recruited a limited number of health workers to strengthen its surveillance system and emergency response capability and to cater for the needs of Displaced Syrians living in the informal tented settlements, in addition to a limited number of administrative employees at central and peripheral levels. A total of 92 new staff was recruited in PHC, in dispensaries’ and in public hospitals. Due to financial constraints, however, this number diminished gradually to 42 by the end of 2015. Retention of the remaining staff will largely depend on the evolution of the crisis.

Embedded in each program of work were a set of monitoring and evaluation tools that made the stretched activities run smoother. Each program approached the crisis as if they were dealing with an enlarged population of 30%. Immunization activities, PHC services, and secondary care provision were all maintained and effectively expanded while monitoring and maintaining quality standards.

During the crisis, the participation of the private sector and civil society, and networking with different donors, international stakeholders and UN agencies was not only important for health system governance but also for the development of multi–sectoral health strategies. Examples of successful partnerships included the engagement of the primary health care national network and private hospitals in health care delivery to mount a unified and effective response [37].

**Service provision.** Provision of health care has been sustained at all levels throughout the crisis. Primary health care centers and hospitals from both public and private sectors have remained operational. Health programmes, such as those for epidemiological surveillance, immunization, medication for chronic illnesses, tuberculosis, HIV/AIDS and reproductive health, among others, are functioning effectively [38]. Other programs, such as the accreditation of primary health care centers and integration of non–communicable disease management within primary health care, progressed as planned despite the crisis [38].

Nationwide vaccination campaigns for polio and measles have been routinely conducted as needed, and services provided to all those in Lebanonirrespective of nationalities [39]. These immunization campaigns were conducted in accordance with the district physicians, municipalities, civil society and schools. Community health workers, including volunteers from universities and schools, participated in the door–to–door immunization campaigns [38]. Additionally, the epidemiological surveillance program was able to sustain and even enhance its functions, including measurement and monitoring disease burden, detecting outbreaks, investigating emerging infections and implementing early warning and response system [4]. Staff trainings were conducted by MOPH health experts and adequate precautionary measures were taken at airports and seaports against pandemic threats, such as Ebola and MERS Coronavirus [39,40].

In addition to the primary care centers across Lebanon that were providing health services for Syrians refugees and the public health response, at the hospital level, UNHCR contracted with public and private hospitals to provide for registered Displaced Syrians selected secondary care services, covering 75% of the fees [41]. The additional services financed by UNHCR enabled the MOPH to maintain the functioning of existing units to meet the needs of displaced Syrian refuges while allocating additional dedicated health workers for those living in informal tented settlements [6].

### Health system outputs and outcomes

**Health service utilization.** Since 2013, the number of primary health care centers in the national network as well as the number of beneficiaries steadily rose [38]. In 2014, the total beneficiaries of the primary health care network exceeded 1.2 million, compared to about 700 000 in 2009 [38]. These beneficiaries include both Lebanese and Syrian nationals. In 2014, Syrian nationals made up around 35% of the beneficiaries for the primary health care national network [38].

**Figure 1:**
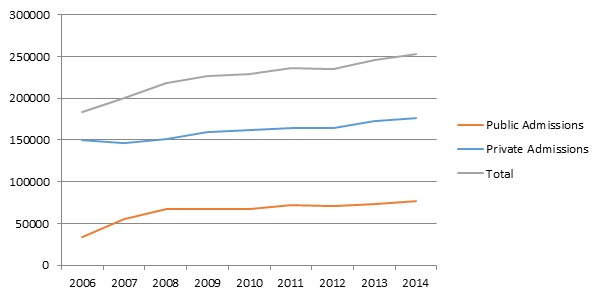
Hospital admissions by 2006–2014.

**Figure 2:**
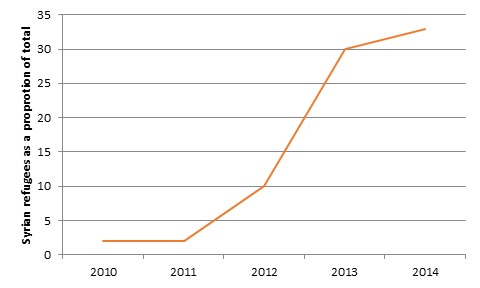
Utilization of hospital services in public hospitals 2010–2014.

Private and public hospitals continue to deliver quality services. While the MOPH has sustained its coverage of hospital admissions for uninsured Lebanese, admissions for insured Lebanese have not been disrupted by the Syrian refugee crisis [1] (**Figure 1**). Meanwhile, the proportion of Syrian beneficiaries in Rafic Hariri Governmental University Hospital has continued to increase from 2% in 2010 to 33% in 2014 (**Figure 2**) [42].

**Figure 3:**
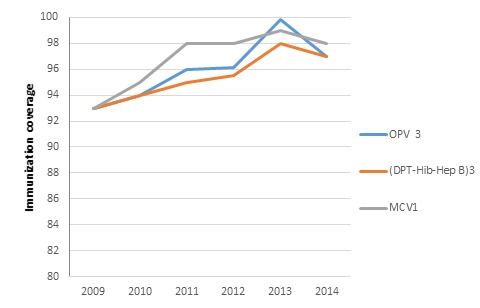
Polio, DPT (diphtheria, pertussis and tetanus) and measles vaccination rates in Lebanon, 2009–2014.

In terms of immunization coverage, vaccination rates for measles and diphtheria, pertussis and tetanus (DPT) are considered important indicators of health system performance [43]. The vaccination campaigns achieved high vaccination rates for both Lebanese and Syrian beneficiaries [38] (**Figure 3**).

**Health expenditures.** To ensure uninterrupted financial coverage, the MOPH developed and implemented a reform strategy in 1998 to rationalize health expenditures targeting the high financial burden on households. This strategy resulted in lower out of pocket expenditures, which declined from 60% in 1998 to 34% in 2013, at the peak of the Syrian crisis [2,35,44].

**Morbidity and mortality.** Despite political instability, Lebanon has achieved the health related Millennium Development Goals (MDGs 4, 5 and 6) [7]. Infant Mortality Rate (IMR) fell sharply from 33.5 per 1000 live births in 1996 to 8 per 1000 live births while Under 5 Mortality Rate (U5MR) declined from 36.5 per 1000 live births in 1996 to 9 per 1000 live births in 2013 [45]. Similarly, the maternal mortality ratio decreased from 140 per 100 000 live births in 1990 to 16 in 2013 [45–47]. Child and Maternal Mortality Observatory data confirm the downward trend in these indicators over the recent years [1,45,46].

**Prevention of outbreaks of infections.** The influx of Syrian refugees has increased the risk and exposure to communicable diseases, including those that previously did not exist in Lebanon [23]. Communicable diseases, such as polio, measles and waterborne infections, are considered the greatest public health risks in refugee situations [48]. Outbreak prevention and control, therefore, represent an important measure of the resilience of a health system.

Lebanon effectively managed several outbreaks including for measles. In 2013, the number of reported measles cases was 1760, compared to 29 cases in 2015 [49]. The spread of Leishmaniasis, an infection which was previously not noted in Lebanon, was also avoided despite the existence of its vector, the sand fly, in North Lebanon and the Bekaa, and the presence of infected Syrians as a human reservoir [49]. The number of Leishmaniasis cases fell substantially between 2013 and 2015 (1033 to 32 cases), with only three Lebanese citizens contracting the disease during the crisis [49]. Additionally, Lebanon was able to stay polio–free, despite reemergence of the disease in Syria [38]. The MOPH ensured that the vaccination campaigns reached the maximum number of children by conducting school field visits, by having an MOPH vaccination team at every UNHCR refugee registration entry point, by coordination with the MOPH officers at district level, and primary care centers and by providing door–to–door coverage. Syrian refugees have also received routine immunizations and other vaccinations, such as polio and measles through the vaccination campaigns spearheaded and coordinated by the MOPH, UNICEF and WHO. As for cholera, and despite it being considered a public health threat in Lebanon by WHO due to the refugee crisis, Lebanon was cholera–free from 2013 to 2015 [49].

**Figure 4:**
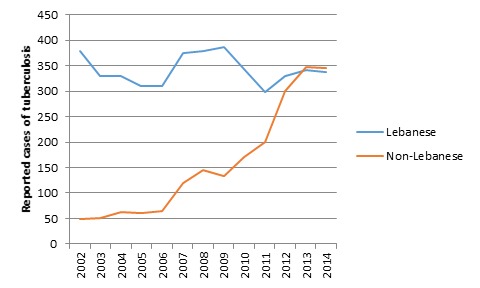
Reported cases of tuberculosis in Lebanon among Lebanese and Non–Lebanese persons 2002 to 2014.

Case notification rate of tuberculosis (TB) in Lebanon had been declining until 2011. In 2012, the case notification rate increased by 27%, however [50] (**Figure 4**). This increase was attributed to a rapid rise in the number of Syrian refugees, as only 48% of notified TB cases was among non–Lebanese [50]. Early detection, isolation, and treatment of TB cases in specialized centers and hospitals among the displaced populations prevented an outbreak in host communities [51]. In 2014, the treatment success rate was 76%, with one half the TB cases receiving treatment completely cured [51].

## Discussion

Our findings indicate that the health system in Lebanon was able to maintain service delivery for both refugees and Lebanese citizens, prevent communicable diseases and sustain improvements in morbidity and mortality levels in the presence of major external and internal shocks, despite relatively limited increase in system inputs. The health system was “able to adapt to change and retain functionality” of governance, financing and service delivery “while maintaining achievements” [26–28]. As the crisis evolves, the resilience of health care service delivery in Lebanon will be continuously monitored, as the health system comes under increasing pressure.

The resilience of the Lebanese health system could be attributed to four major factors. First, networking with the multitude of partners in the health sector [52] and the mobilization and support of regional and global partners, were at the core of the response to the Syrian refugee crisis. This integrated approach was evident in the refugee response plan that was developed by key actors and implemented by a wide array of service providers, including from the private and public sectors and NGOs [25]. Additionally, the Lebanese health system was able to draw upon diverse sources of funding and multiple conduits for service delivery. Although multiple financing sources and service providers can lead to fragmentation, good governance based on a public private partnership helped secure a constant stream of funds, primarily through both reallocation of resources and internal resource mobilization, which allowed patients to bypass government bureaucracy and partially compensate for the delayed and scarce international aid. Integration and smart dependency achieved in Lebanon is a key feature of resilient health systems [25].

Second, adequate infrastructure and sufficient supply of health human resources was vital in absorbing the additional numbers of refugees. Resilient health systems have the ability to tap in to excess capacities for an optimal health response during a crisis [25,53]. Lebanon, which had a diverse set of providers also had an oversupply of hospital beds and technology that was used to meet the increased demand during the crisis [2,54]. An adequate supply of a committed and responsive workforce is a precondition for resilience [25,53]. In Lebanon, the health workforce is well accustomed to crisis situations [55]. This experience ensured a regular supply of health human resources that catered to both refugee and Lebanese populations.

Third, a comprehensive communicable disease response helped combat outbreaks, a major health priority during a refugee crisis [48]. The Ebola crisis in West Africa highlighted the importance of epidemiological surveillance as part of an “aware” system in outbreak control [25,53]. In Lebanon, the primary health care department, along with the epidemiological surveillance unit, played an important role in ensuring effective and ongoing surveillance. Widespread immunization campaigns, with augmented community engagement activities, were employed in a timely manner and synchronized with regional levels to achieve high coverage rates. Effective immunization coverage, coupled with the early warning and response system, allowed for prevention and control of the spread of communicable diseases.

Fourth, integration of refugee health care within the national health system, made possible through the settlement of refugees within Lebanese communities rather than camps, was also an important factor. Although this approach may have been problematic for the host communities, it reduced administrative and set–up costs, and enabled more responsive service delivery. It also shifted the burden to several geographic areas in Lebanon and to several different players in the Lebanese health system. The benefits of the integrated health system approach over the approach, which creates multiple parallel service delivery and financing systems, have been documented in other refugee crises [56].

Our findings suggest a resilient response by the Lebanese health system to the refugee crisis. Despite the limited resources and the turmoil caused by the war in Syria, Lebanon has been able to cope with an unprecedented influx of refugees, maintain improvements in mortality and morbidity outcomes in the country and achieve the MDG targets.

Our observations in a real empirical setting lead us to suggest a revised definition of resilience of health systems: “Resilience is the capacity of a health system to absorb internal and external shocks, and maintain functional health institutions while sustaining achievements.” We believe that this revised definition describes a real life and tested experience of resilience in an unprecedented setting.

We identified four major factors that enabled resilience: (i) networking with stakeholders (ii) diversification of the health system that provided for adequate infrastructure and health human resources (iii) a comprehensive communicable disease response and (iv) the integration of refugees into the health system. A question that remains unanswered is the longer–term sustainability of the current response. Although, thus far, Lebanon has sustained achievements in morbidity and mortality levels, the magnitude and the chronic nature of the crisis continues to pose a threat to the health system.

The study has three main strengths. First, to our knowledge, this is the first study to investigate the resilience of a health system during an ongoing major refugee crisis. Second, the use of the input–process–output–outcome model to analyze the data and to categorize the health system resilience has helped to frame the system as a whole, and shed light on the possible contributing factors to achieving resilience. Third, the study used multiple sources of information, including the public, private, civil society and humanitarian sectors, to provide a comprehensive view of the Lebanese health system.

Several limitations are also acknowledged. First, the literature lacks a rigorous and scientifically validated method for measuring and proving resilience in health systems. We used a model that included several dimensions of resilience identified from published and gray literature, in addition to health system performance indicators which we considered to be important measures relating to resilience. Second, the study was limited by the availability of data on the dispersed refugee population and the ongoing influx of refugees. Third, the dynamic nature of the refugee situation means data need to be regularly updated.

Notwithstanding limitations, however, our study contributes to an area of global importance and helps empirically to illuminate effective response from a health system that has shown resilience in spite of the most severe refugee crisis of recent times experienced anywhere in the world.
